# Obstructed hemivagina with ipsilateral renal agenesis (OHVIRA) syndrome: Typical presentation of a rare syndrome

**DOI:** 10.1016/j.radcr.2023.04.016

**Published:** 2023-05-11

**Authors:** Bhavana Devanabanda, Bradley Chatterton, Irfan Nazir Hassan, Jay Patel

**Affiliations:** Department of Radiology, Cooperman Barnabas Medical Center, Rutgers RWJ Barnabas Health, 94 Old Short Hills Road, Livingston, NJ 07039, USA

**Keywords:** OHVIRA syndrome, Herlyn-Werner-Wunderlich Syndrome, Mullerian aplasia

## Abstract

Obstructed hemivagina with ipsilateral renal agenesis (OHVIRA) syndrome is a rare congenital malformation of the Mullerian duct. We report on a 34-year-old female who presented to the emergency department for cramping lower abdominal pain and pelvic pressure with vaginal spotting. Physical exam showed marked swelling in the right adnexa, and laboratory exams were within normal limits except for positive Coronavirus disease 2019 (COVID-19) status. Transvaginal ultrasound revealed 3 well circumscribed, round hypoechoic complex cystic lesions with arterial doppler detected within the peripheral walls. Magnetic resonance imaging of the abdomen and pelvis showed a right hemivagina, right hematosalpinx, right hematometra and right renal agenesis, compatible with OHVIRA syndrome. The patient was informed of elective surgical procedure but was unable to undergo surgery at this time secondary to COVID status. The patient was therefore recommended oral contraceptive therapy for suppression of menses and protection of endometrial lining.

## Introduction

Uterine didelphys, a unilateral obstructed hemivagina with an ipsilateral renal anomaly is the classic presentation of obstructed hemivagina with ipsilateral renal agenesis (OHVIRA) syndrome, which is otherwise known as Herlyn-Werner-Wunderlich syndrome [1,2]. This condition is a very rare congenital abnormality, which occurs secondary to aberrant Mullerian duct and Wolffian duct development [1,3]. The incidence of OHVIRA syndrome is unknown, and only a few case reports have been published. Here, we will examine the findings apparent on multiple imaging modalities, which aid in the diagnosis of OHVIRA syndrome.

## Case presentation

A 34-year-old female presented with complaints of vaginal spotting, cramping lower abdominal pain and pelvic pressure. On physical exam the patient had normal appearing external genitalia, single vaginal introitus. On digital examination, marked swelling was appreciated in the right adnexa. Patient's vitals and other laboratory parameters were within normal range. The patient experienced menarche at 13 years of age but afterwards experienced menstrual irregularity. She was afterwards diagnosed with Kallman Syndrome and received hormone replacement therapy of Estradiol Patch (0.05 mg) and Provera (2.5 mg daily).

**Fig. 1:**
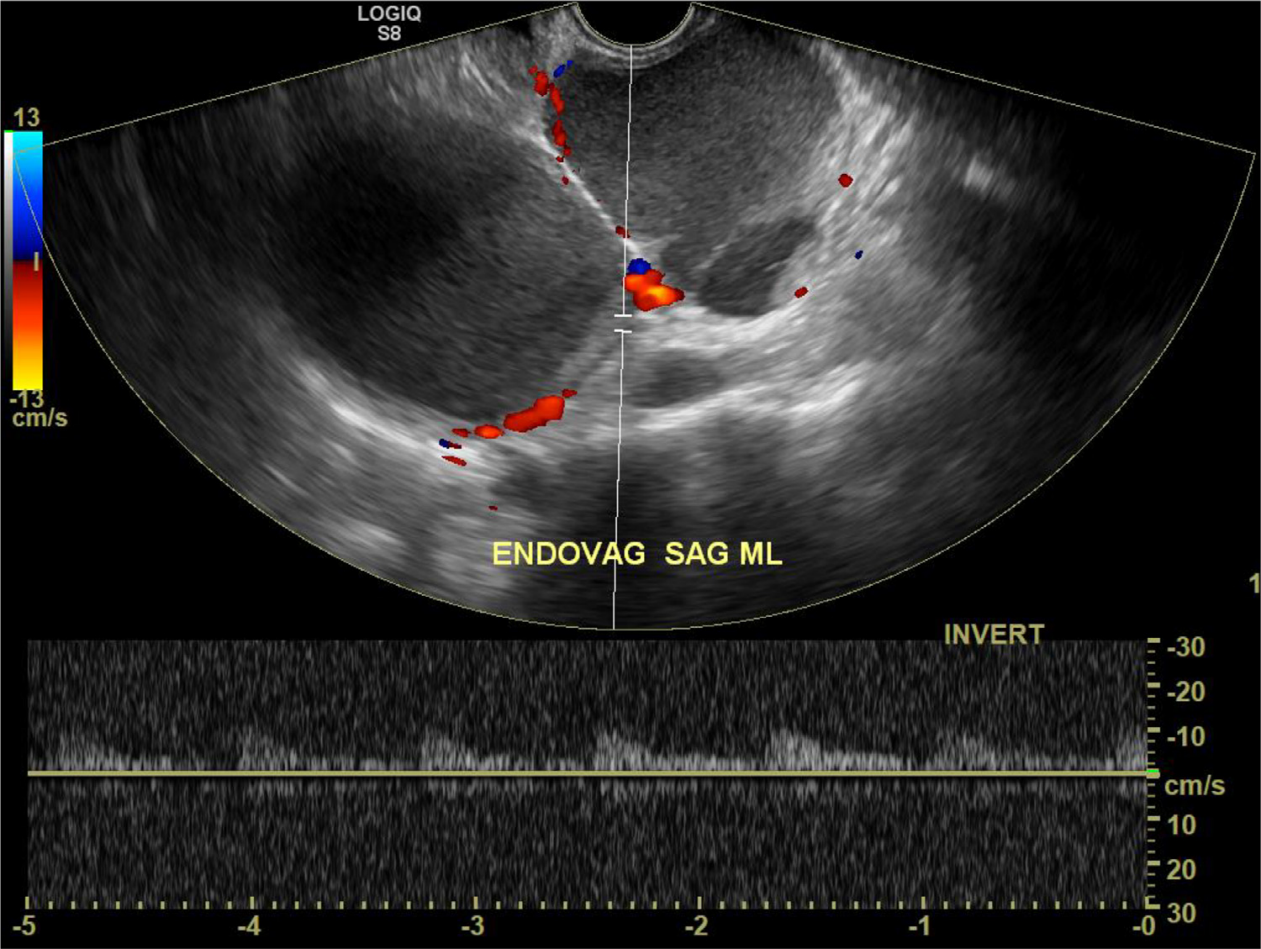
Transvaginal Ultrasound. Three well circumscribed, round, hypoechoic lesions are identified. The lesions contain complex fluid. Eco-color Doppler detected arterial-like blood flow within the walls of the lesion.

**Fig. 2:**
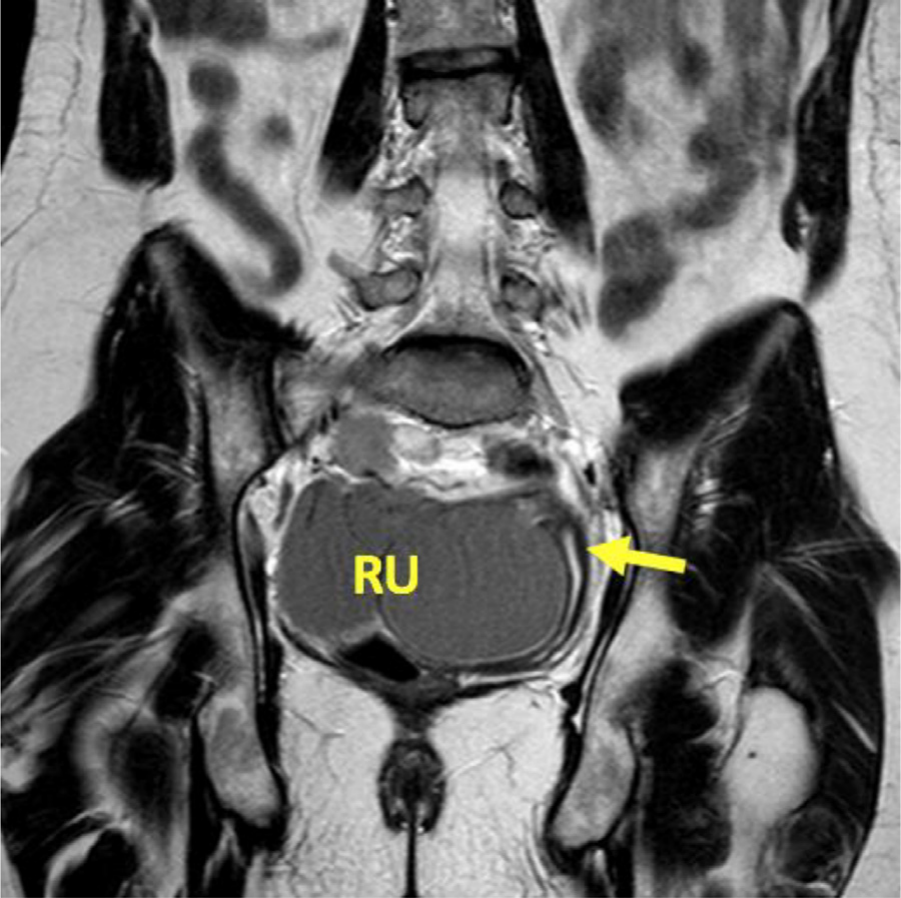
MRI T2**-**Weighted coronal image. Distended right uterus with blood products (RU) and collapsed left hemivagina (arrow).

Transvaginal ultrasound revealed three well circumscribed, round, hypoechoic complex cystic lesions. Eco-color Doppler showed presence of arterial-like blood flow within the peripheral walls of the lesion (Fig. 1). Magnetic resonance imaging (MRI) of the abdomen and pelvis was subsequently obtained for further evaluation. Patient was noted to have uterus didelphys, right hemivagina, right hematosalpinx, right hematometra and right renal agenesis (Figs. 2–Fig. 4, Fig. 5). The left hemivagina, uterus, fallopian tube and kidney were normal. Patient was diagnosed with OHVIRA syndrome.

The patient was informed that there is an elective corrective procedure, however the patient was unable to undergo the procedure at this time secondary to positive COVID status. The patient was recommended continuous oral contraceptive pill (OCP) therapy for suppression of menses and protection of endometrial lining, which she accepted. The patient was provided extensive instruction regarding OCP and pain control regimen.

## Discussion

Obstructed hemivagina with hematometrocolpos, hematosalpinx, didelphys uterus and ipsilateral renal anomaly is the classic presentation of OHVIRA syndrome, otherwise known as Herlyn-Werner-Wunderlich Syndrome [1]. It is a very rare congenital abnormality and the true incidence rate is unknown, however estimated between 0.1% and 3.8% [2]. It is caused by an anomalous development of the paramesonephric (Mullerian) duct and the mesonephric (Wolffian) duct [1,3]. The Mullerian duct is embryonic structure which typically develops into the oviduct, uterus, cervix and upper vagina [4]. The Wolffian ducts act to induce development of the Mullerian duct, as well as initial development of the renal system. Abnormalities of the Wolffian ducts therefore result in anomalous Mullerian ducts, causing unilateral renal agenesis and an imperforate hemivagina apparent in OHVIRA syndrome [3].

OHVIRA syndrome can be further subdivided based on the extent of hemivaginal obstruction.

1. Type 1 is defined as having a completely obstructed hemivagina.
  1.1. Completely obstructed hemivagina with blind hemivagina
  1.2. Completely obstructed hemivagina with cervicogenic atresia without communicating uteri
2. Type 2 is defined as having an incompletely obstructed hemivagina
  2.1. Incompletely obstructed hemivagina with partial reabsorption of the vaginal septum
  2.2. Incompletely obstructed hemivagina with communicating uteri [2]

Type 1 is associated with an earlier age of symptomatic onset and diagnosis. There is also a shorter duration in the onset of cyclic pelvic pain for Type 1. However, the incidence of dysmenorrhea, occasional mucopurulent discharge and vaginal hemorrhage is increased for patients with Type 2. Patients within Type 1 also have an increased risk of hematosalpinx and hemoperitoneum and typically present with abdominal pain several months following menarche [2,5]. However, patients with Type 2 typically present with purulent or bloody vaginal discharge and ascending genital system infection years after menarche and typically develop pain several years following menarche [5].

**Fig. 3:**
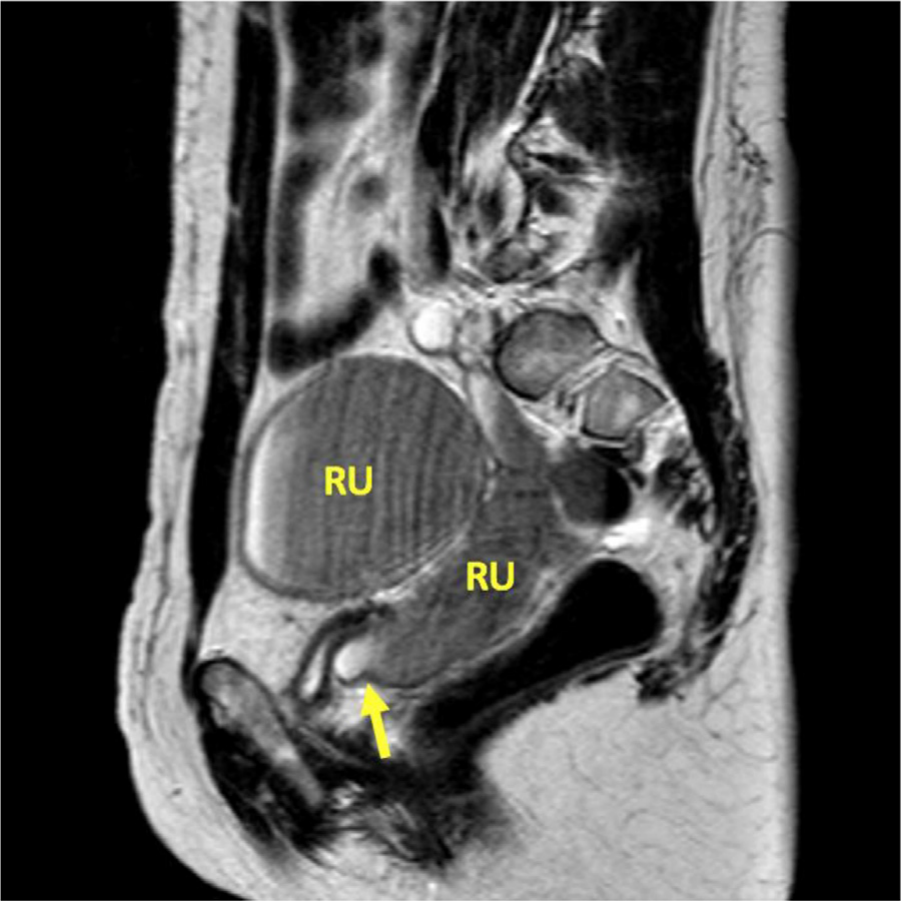
MRI T2**-**Weighted sagittal image. Transverse membrane (arrow) obstructing the right hemivagina.

Our patient fits the criteria of Type 1: As demonstrated in Figure 3, there is a membrane obstructing the right hemivagina. The large distended right uterus is a result of chronic accumulation of blood caused by repeated menstrual cycles over the years. The distended right hematometra has compressive effects on the left uterus as seen in Figure 2.

**Fig. 4:**
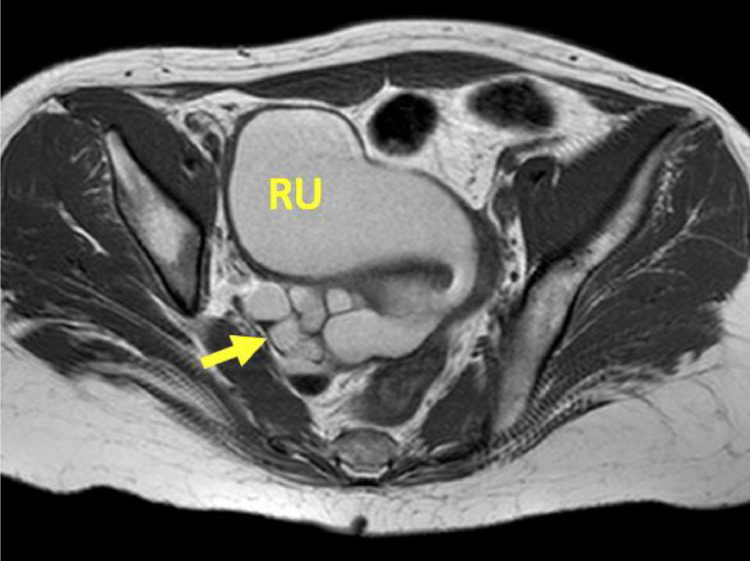
MRI T1-Weighted axial. Large serpiginous, tubular structure (arrow) in the right adnexal region with septa representing a dilated right fallopian tube. There are dependent T1 hyperintense products and hypointense fluid levels in the endometrial cavity and fallopian tube compatible with chronic blood products.

**Fig. 5:**
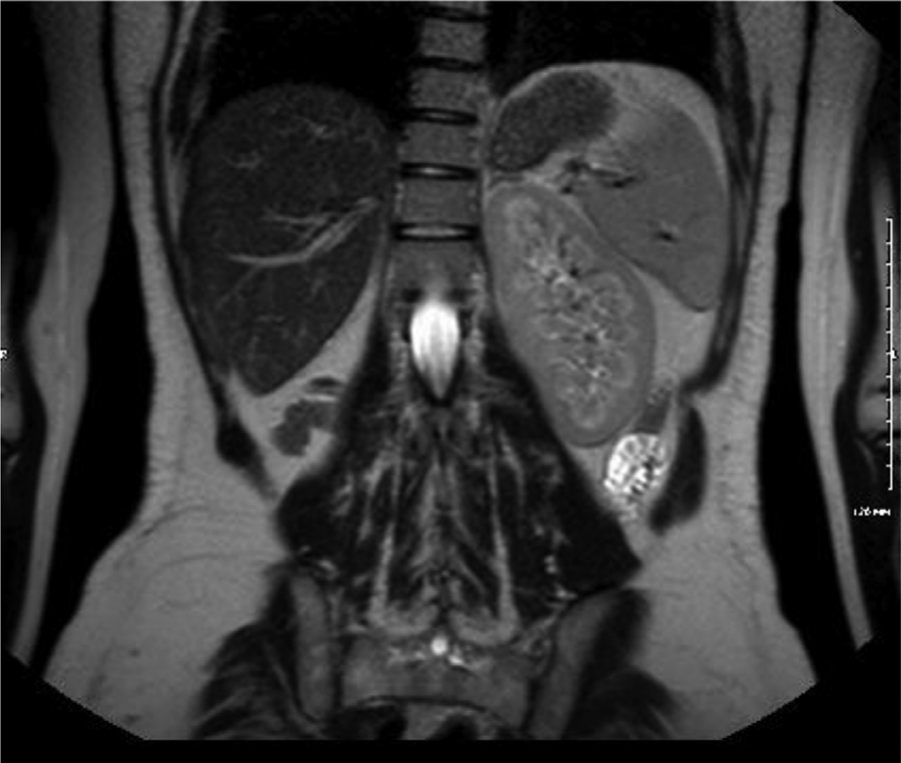
MRI T2**-**Weighted coronal. Right kidney is not visualized. There is mild compensatory hypertrophy of the left kidney in the setting of right renal agenesis.

OHVIRA syndrome can present with a variety of symptoms. The most common symptoms include lower abdominal pain, dysmenorrhea, and a tender vaginal mass [5]. Other common symptoms include recurrent urinary tract infections, urine retention, vaginal discharge and chronic pelvic pain [6]. Patients may also present with a variety of other anomalies, including renal dysplasia, an ectopic ureter or ovarian mispositioning [7]. Anomalies of the right side occur two times more frequently when compared to the left side [5,8]. OHVIRA syndrome is also associated with endometriosis if not diagnosed and treated early [8,9]. Several cancers have also been described in patients with OHVIRA syndrome previously. This includes adenocarcinoma of the obstructed uterine cervix as well as clear cell carcinoma of the obstructed hemivagina [9]. The major concern with OHVIRA syndrome is the preservation of fertility.

Women with didelphys uterus have a high likelihood of becoming pregnant, where about 80%-87% may be able to conceive and about 62% were delivered at term [5,9]. However, these patients also have elevated rates of premature delivery compared to the normal population (22%) [9]. Therefore, cesarean sections are necessary for approximately 80%-84% of patients with OHVIRA syndrome [5,9]. The most common indication for cesarean section was breech position, which was reported in 51% of patients [5].

Early diagnosis is crucial to prevent further complications associated with OHVIRA syndrome. Transvaginal ultrasonography is a low cost method and can be utilized to examine the adnexa and uterus without radiation exposure [2,8]. Computed tomography can also be utilized in the diagnosis of OHVIRA syndrome, however MRI is considered to be the gold standard [9]. MRI provides further evaluation of uterine morphology, can detect communication between vaginal and uterine lumen, and can better characterize fluid content [8].

Surgical intervention is the most effective method to retain fertility and alleviate symptoms in patients with OHVIRA syndrome. Surgical intervention for Type 1.1, 2.1 and 2.2 is directed at resecting the obstructed vaginal septum, and most patients can recover completely following the resection. The optimal surgical timing is at the time of menstruation, because a large distended hematocolpos can be visualized and aid in resection [2]. Laparoscopic technique can be utilized for adhesion lysis and resection of endometriosis if present [10]. Surgical management for a patient with Type 1.2 differs from the other types because of the presence of cervical atresia. It is recommended that patients with this type of OHVIRA syndrome have a laparoscopic or transabdominal resection of the affected ipsilateral uterus [2,7]. If the patient cannot undergo surgery immediately, oral contraceptives can be used to suppress menstruation and provide symptomatic relief [11].

## Conclusion

OHVIRA is a rare congenital syndrome caused by anomalies in the development of Mullerian and Wolfian ducts. The obstructed hemivagina can cause a wide range of complications from cyclic abdominal pain during menses, endometriosis, infertility and sepsis. MRI is the gold standard for diagnosis and classification of hemivaginal obstruction. Surgical resection of the obstructed hemivagina is the gold standard for management. Early detection and intervention is important in improving quality of life and preventing long term complications.

## Patient consent

We would like to thank the patient for allowing us to write this case. All Identifiers were removed while writing this report.
